# *Aerococcus urinae*, a rare cause of aortic root abscess: a case report

**DOI:** 10.1186/s13256-022-03564-8

**Published:** 2022-11-17

**Authors:** Chong Wei Tiong, Caroline Bartolo, Aaron Walton, Eugene Athan

**Affiliations:** 1 Department of General Medicine, Barwon Health, Geelong, VIC Australia; 2 Department of Infectious Diseases, Barwon Health, Geelong, VIC Australia; 3 Deakin University, Melbourne, VIC Australia

**Keywords:** Endocarditis, Aerococcus, Bacteraemia, Case report

## Abstract

**Background:**

*Aerococcus urinae* is a bacterium of emerging clinical interest that most commonly causes urinary tract infections (UTI) but can also result in invasive infections. It is a catalase-negative, alpha-haemolytic gram-positive coccus that grows in clusters or tetrads and usually causes urinary tract infections. While rare, infective endocarditis must be considered when *A. urinae* is isolated in blood culture. The mortality rate of *A. urinae* infective endocarditis is similar to overall endocarditis mortality. We report a rare case of aortic root abscess caused by *A. urinae.*

**Case presentation:**

An 82-year-old Caucasian man presented to hospital with behavioural change and severe malnutrition and was managed for psychotic depression. On day 34 of his inpatient stay, a febrile episode prompted blood cultures, which grew *Aerococcus. urinae*. Investigations revealed a bicuspid aortic valve, aortic valve endocarditis and aortic root abscess. He also had prostatomegaly. He underwent aortic valve replacement, received 6 weeks of intravenous ceftriaxone and recovered.

**Conclusion:**

Infective endocarditis should be considered in patients with persistent *Aerococcus urinae* bacteraemia. Accurate identification with mass spectrometry is recommended to avoid misidentification as staphylococcus, streptococcus or enterococcus, which is a possibility with conventional laboratory methods.

## Background

*Aerococcus urinae* is a bacterium of emerging clinical interest that most commonly causes urinary tract infections (UTI) but can rarely result in invasive infections. It is a catalase-negative, alpha-haemolytic gram-positive coccus, which grows in clusters or tetrads [1, 2]. It accounts for an estimated 0.15–0.8% of all UTI cases [3, 4]. However, this is likely an underestimate because *A. urinae* has often been misclassified as *Staphylococcus, Streptococcus* or *Enterococcus*, due to similarities on microscopy and colony reaction to hemolysis [1, 5]. While rare, infective endocarditis must be considered when *A. urinae* is isolated in blood culture [1]. To the best of our knowledge, this is the third reported case in the English literature and the first reported case in Australia of aortic root abscess caused by *Aerococcus urinae*.

## Case report

**Fig. 1:**
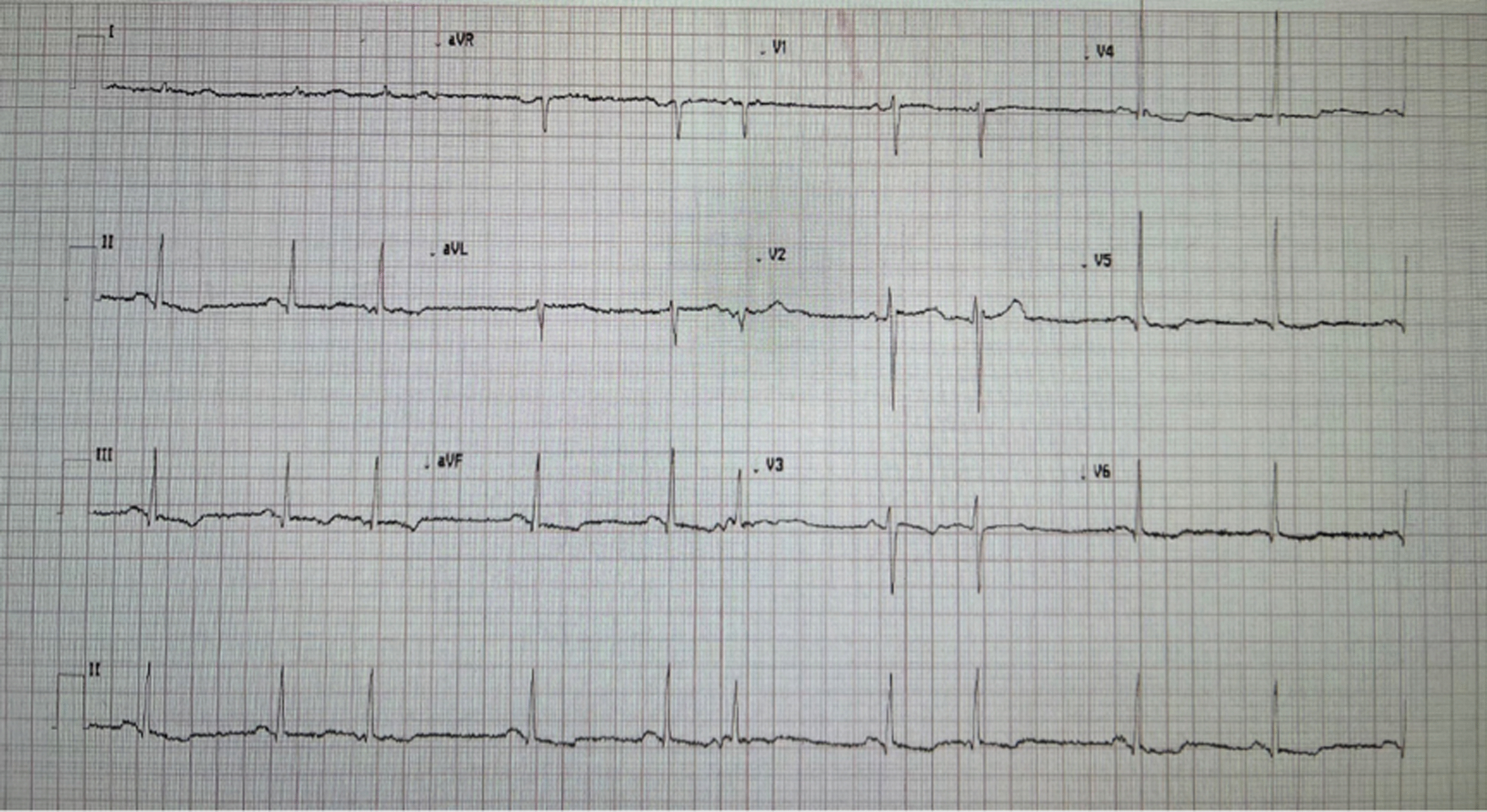
Electrocardiogram at admission

**Fig. 2:**
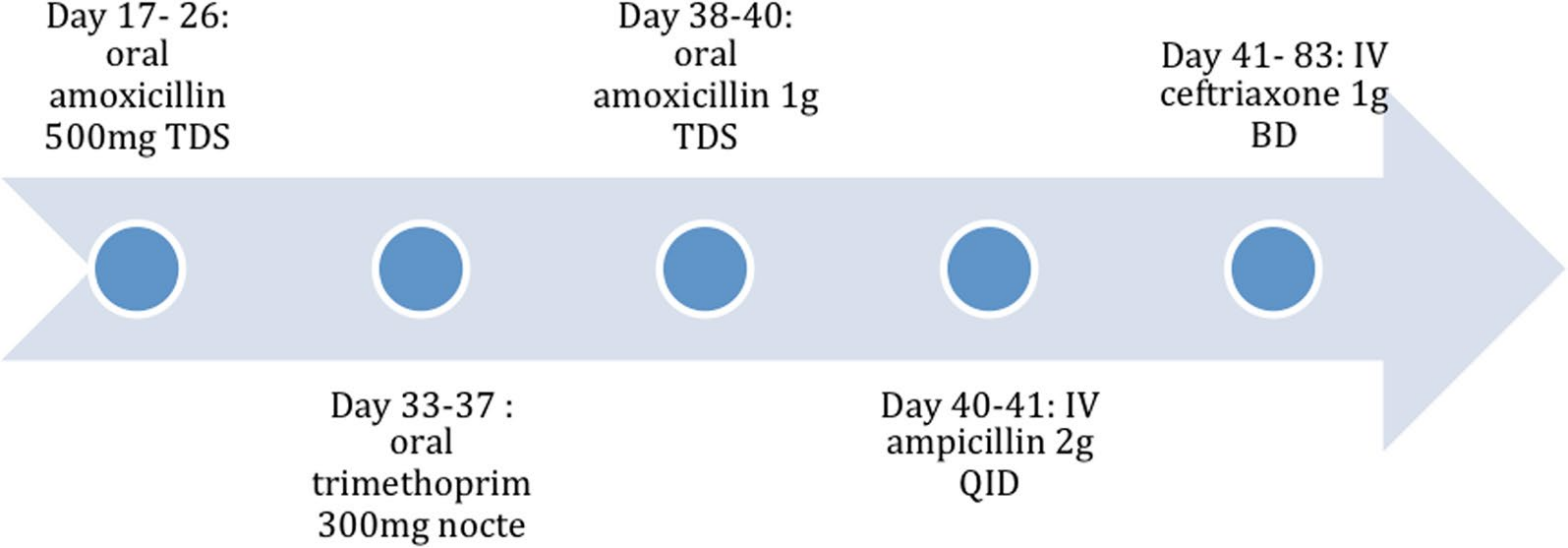
Summary of antibiotics given during this hospital admission

**Fig. 3:**
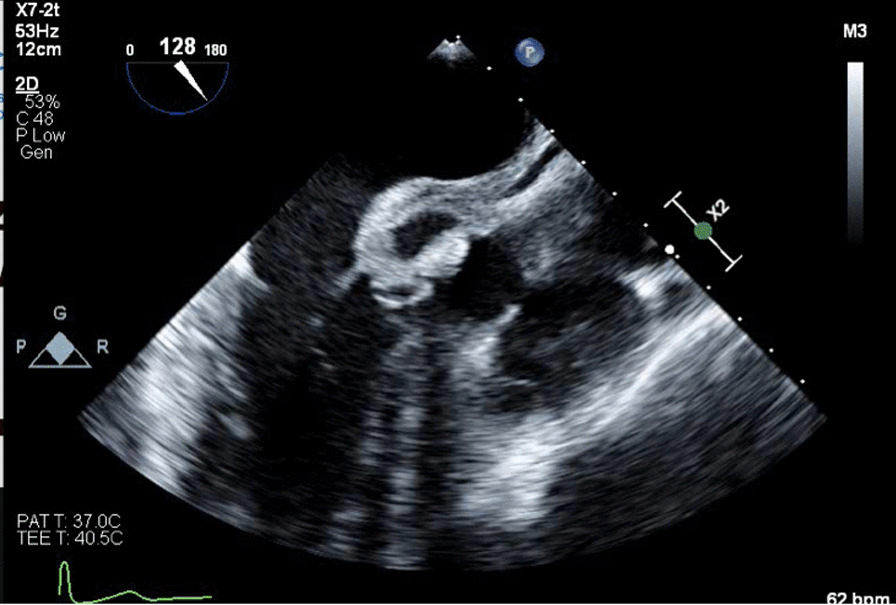
Transesophageal echocardiogram demonstrating aortic root abscess

An 82-year-old Caucasian man was admitted to a regional tertiary hospital for low mood and behavioral change for 2 weeks, as well as reduced appetite, malnutrition and weight loss. His past medical history was unremarkable other than chronic lower urinary tract symptoms, and he was not taking any regular medications. He is a retired engineer who is independent with his activities of daily living. He is also the main caregiver for his daughter who has been diagnosed with brain cancer. His wife passed away a year ago. He does not have significant alcohol use and does not use recreational drugs. He has a distant smoking history, last smoked 50 years ago. On admission his blood pressure was 108/70 mmHg. His heart rate was 70 beats per minute. His oxygen saturation was 98% on room air. His respiratory rate was 19 breaths per minute. He was afebrile at 36.3 °C. He was oriented to time and place, and performed serial sevens calculations without difficulty. Speech was slow and affect was flat. Cardiovascular examination was normal with dual heart sounds, no murmur and no clinical evidence of heart failure. Respiratory and abdominal examination was normal. Cranial nerve examination was unremarkable. Neurological examination of upper limbs and lowers limbs was unremarkable, with normal tone, power, coordination, sensation and reflexes. A dental examination was not performed. Blood tests showed a normal white cell count (WCC) 6.2 × 10^9^/L (reference range: 4–11 × 10^9^/L) and C-reactive protein (CRP) < 2.9 mg/L (< 2.9 mg/L). Figure 1 shows his electrocardiogram at admission. Importantly, there was no atrioventricular (AV) block. Magnetic resonance imaging of his brain showed small old lacunar infarct in left lentiform nucleus, which would not explain his presentation. He was reviewed by the psychiatry team, who diagnosed severe psychotic depression and commenced the patient on citalopram and risperidone. On day 9 of his hospital stay, he developed acute urinary retention, requiring indwelling urinary catheter placement. A renal tract ultrasound revealed moderate prostatomegaly. He remained in hospital for management of his severe malnutrition and monitoring for re-feeding syndrome. He had two episodes of presumed catheter associated urinary tract infection with growth of *Enterococcus faecalis* and *Escherichia coli*, and received courses of oral amoxicillin 500 mg TDS and oral trimethoprim 300 mg daily respectively. On day 34, a febrile episode prompted blood cultures. He had no localizing symptoms. On examination there were no heart murmurs, peripheral stigmata of infective endocarditis, or signs of heart failure. After 25 hours, both aerobic and anaerobic blood culture bottles flagged with gram-positive cocci in clusters resembling Staphylococci, which were eventually identified by mass spectrometry as *Aerococcus urinae.* Susceptibility testing was performed using Etest with breakpoints as described by Clinical and Laboratory Standards Institute (CLSI) criteria. The penicillin and ceftriaxone MICs were 0.064 µg/mL and 0.50 µg/mL respectively (both susceptible). Urine culture was sterile. A repeat WCC was 5.5 × 10^9^/L (4–11 × 10^9^/L) while CRP increased to 73 mg/L (< 2.9 mg/L). See Table 1. Hepatitis B surface antigen,hepatitis C antibody, and Human Immunodeficiency Virus (HIV) antigen/antibody was not detected. *Treponema pallidum* Immunoglobulin G (Ig) was non-reactive. The patient was commenced on intravenous ceftriaxone 1 g twice a day. A summary of the antibiotics administered is shown in Fig. 2. He remained bacteraemic after 6 days so a transthoracic echocardiogram was performed which showed a bicuspid aortic valve and mobile densities on both mitral and aortic valves as well as aorto-mitral curtain thickening. A transesophageal echocardiogram (TOE) subsequently showed a cavity (1.6 cm × 0.9 cm) at the aorto-mitral curtain, consistent with an abscess (Fig. 3). The patient proceeded to have an aortic valve replacement and patch repair of the left coronary sinus, which had been destroyed by the abscess. Operative specimens were sterile however the operation had been delayed due to the patient’s initial reluctance to proceed with surgery and he had received antibiotics for over 2 weeks by the time surgery was performed. He received a total of 6 weeks of ceftriaxone, and was transferred to a subacute rehabilitation centre for re-conditioning.

**Table 1:** Investigation results

	At admission	Day 34 of hospital admission	Normal range
Haemoglobin (g/L)	157	128	130–180
White cell count (× 10^9^/L)	6.2	6.9	4–11
Neutrophils (× 10^9^/L)	4.2	5.2	2–8
Lymphocytes (× 10^9^/L)	1.4	1.1	14
Monocytes (× 10^9^/L)	0.5	0.4	< 1.1
Eosinophils (× 10^9^/L)	< 0.1	< 0.1	< 0.6
Basophils (× 10^9^/L)	< 0.1	< 0.1	< 0.3
Platelets (× 10^9^/L)	227	257	150–450
Sodium (mmol/L)	139	132	135–145
Potassium (mmol/L)	4.2	4.5	3.5–5.2
Chloride (mmol/L)	103	94	95–110
Bicarbonate (mmol/L)	29	29	22–32
Anion gap (mmol/L)	11	14	9–19
Urea (mmol/L)	7.7	6.8	3.5–11
Creatinine (µM/L)	95	80	60–110
eGFR (mL/min)	64	79	> 59
Adjusted calcium (mmol/L)	2.16	2.12	2.15–2.55
Magnesium (mmol/L)	0.86	0.76	0.7–1.1
Phosphate (mmol/L)	1.14	0.81	0.75–1.5
Total protein (g/L)	65	51	60–80
Albumin (g/L)	38	23	35–50
Globulin (g/L)	27	28	23–39
ALP (U/L)	42	60	40–140
Bilirubin (micromole/L)	17	6	< 25
GGT (U/L)	18	49	< 51
AST (U/L)	19	24	< 41
ALT (U/L)	21	35	< 51
CRP (mg/L)	< 2.9	73.4	< 2.9
TSH (mIU/L)	1.98		0.50–5.00

*Hb* Haemoglobin, *WCC* White cell count, *eGFR* estimated glomerular filtration rate, *ALP *alkaline phosphatase, *GGT* gamma-glutamyl transferase, *AST* aspartate aminotransferase, *ALT* alanine transaminase, *CRP* C-Reactive protein, *TSH* Thyroid-stimulating hormone, *mmol/L* millimole per litre, *micromol/L* micromole per litre, *g/L* gram per litre, *L* Litre, *mg/L* milligram per litre, *U/L* Units per Litre, *ml/min* millilitre per minute, *mIU/L* milliunits per litre

Two weeks after completion of antibiotic course, he had persistently fluctuating cognitive state, hence had further blood cultures which had no growth as well as a lumbar puncture, which was unremarkable. Cerebrospinal fluid (CSF) white cell count was 2 × 10^6^/L, erythrocyte count was 380 × 10^6^/L. No organisms were seen and there was no growth on CSF culture. CSF protein was 0.26 g/L (0.15–0.45 g/L), while CSF glucose was 3.4 mmol/L (2.8–4.2 mmol/L). A repeat transthoracic echocardiogram 6 weeks after the surgery showed no evidence of ongoing endocarditis. On review 3 months later, repeat WCC and CRP were 5.1 × 10^9^/L and < 2.9 mg/L respectively. He reported feeling well at review 7 months later, without any dyspnea, dizziness or palpitations. He was advised to have annual echocardiogram for surveillance of valve function.

## Discussion

To the best of our knowledge, this is the third reported case in the English literature and the first reported case in Australia of aortic root abscess caused by *Aerococcus urinae*.

Although infective endocarditis is a rare manifestation of *Aerococcus urinae,* a high index of suspicion of deep-seated infection is required if a patient is bacteraemic, even in the absence of clinical findings such as a new heart murmur, clinical signs of heart failure, or other peripheral stigmata of infective endocarditis, as described in this case. Due to the high clinical index of suspicion given the prolonged bacteraemia, with appropriate investigations, the diagnosis was established leading to appropriate therapy.

*Aerococcus urinae* belongs to the group of organisms called *Aerococcus-like organisms* (ALO). Along with *Aerococcus viridans and Aerococcus sanguinicola*, *Aerococcus urinae* is increasingly being recognized as a causative pathogen in many human infections [5]. *A. urinae* most commonly causes urinary tract infections, however invasive infections such as endocarditis can occur. There are also occasional reports of spondylodiscitis and spontaneous bacterial peritonitis caused by *A. urinae* [6, 7]. Risk factors for *A. urinae* infection are male gender, age greater than 65 years, and underlying urinary tract abnormalities [8]. The virulence mechanisms displayed by *A. urinae* are the formation of biofilm on plastic surfaces and the aggregation of human platelets, which can result in the formation of heart valve vegetations [1].

Early identification of *A. urinae* in cultures can be challenging due to the following reasons. Firstly, *A. urinae* is alpha-haemolytic, producing semi-transparent colonies, hence can be misidentified as *Streptococcus* on culture. Additionally, on Gram stain, *Aerococcus* and *Staphylococcus* share similar appearance, as Gram-positive cocci in clusters. However, unlike staphylococci, *Aerococcus* is catalase negative. Due to these challenges, there are often in delays in accurate diagnosis. However, due to improved diagnostic techniques identification of *A. urinae* has improved [5, 9]. *A. urinae* can reliably be identified with 16s rRNA-gene sequence (the gold standard) and mass spectrometry, but less so with VITEK 2 [9]. In addition, routine urine cultures may not isolate *A. urinae*, because *A. urinae* optimally requires prolonged incubation in 5% carbon dioxide in anaerobic conditions to be isolated in urine culture [6]. In a review by Senneby *et al*. [1], out of 42 patients who had *A. urinae* bacteraemia, only 6 patients had urine culture that were positive for *A. urinae*. 15 others had sterile urine, while the rest had urine culture that grew other organisms. In a large number of patients, *A. urinae* was not isolated from the urine samples despite having obvious UTI symptoms [1].

*Aerococcus urinae* displays in-vitro susceptibilities to penicillin, amoxicillin, cefotaxime, ceftriaxone, doxycycline, linezolid, and vancomycin. There is variable resistance to clindamycin, fluoroquinolones, and erythromycin. *A. urinae* is resistant to sulphamethoxazole, while sensitivities to trimethoprim and trimethoprim–sulphamethoxazole are poorly defined and variable depending on methodology [1, 10, 11]. Hence, clinicians need to be aware that the commonly used antibiotics for empirical treatment of urinary tract infections when isolate on culture is pending may be ineffective for treatment of *Aerococcus urinae* infections. In a study of 30 *A. urinae* isolates, 97% of isolates had a ceftriaxone MIC ≤ 0.5 µg/mL. 100% had penicillin MIC ≤ 0.06 µg/mL and vancomycin MIC ≤ 1 µg/mL. High erythromycin and levofloxacin MICs were identified in 17% and 33% of the *A. urinae* isolates respectively [12]. Two older studies have showed a synergistic killing effect when there is combination therapy of penicillin and an aminoglycoside. A recent larger study identified this synergy in only 7 of 15 isolates [13]. Both the Clinical and Laboratory Standards Institute (CLSI) and the European Committee on Antimicrobial Susceptibility Testing (EUCAST) have published recommended clinical breakpoint MICs for *A. urinae* [14, 15].

The incidence of infective endocarditis among patients with *A. urinae* bacteraemia is unclear. A population-based study reports that of 16 patients with *A. urinae* bacteraemia, 3 patients had infective endocarditis [1]. In a review of 46 cases of *A. urinae* infective endocarditis, mortality was reported as 28.2%, similar to overall endocarditis mortality reported. Of patients who underwent surgery, 80% survived. On review of the patients who had a good outcome, 80% of them received antibiotic therapy for 4–6 weeks. While a majority of individuals with *A. urinae* infective endocarditis had urologic abnormalities, underlying valvular conditions were less frequent [8].

Duration of antibiotics for *A. urinae* infective endocarditis is currently guided by expert opinion. However, the outcomes of previous cases in the literature would suggest the need for further research regarding extended intravenous antibiotic therapy for uncomplicated bacteraemia. Two case reports described patients who failed therapy after a short course (≤ 10 days) of intravenous antibiotics [2, 16].

## Conclusion

There are a few learning points from this case. Firstly, it is important to consider infective endocarditis in patients with *A. urinae* bacteraemia especially when there is persistent bacteraemia. Second, laboratory diagnosis may initially misidentify *Aerococcus* spp. as *Staphylococcus, Streptococcus* or *Enterococcus* from microscopy and culture therefore use of diagnostic modalities such as mass spectrometry for confirmation of identification is recommended as it will allow for more targeted antimicrobial therapy until susceptibility results are available.

## Data Availability

All data generated and/or analyzed during the current study are available from the corresponding author on reasonable request.
